# Morphology of Lymphoid Tissue in the Lungs of Guinea Pigs Infected with *Mycobacterium bovis* against the Background of Vaccine Immunity and the Action of Betulin and Its Derivatives

**DOI:** 10.3390/vaccines10122084

**Published:** 2022-12-06

**Authors:** Ivan N. Koshkin, Vasily S. Vlasenko, Valentina I. Pleshakova, Larisa E. Alkhimova, Andrey V. Elyshev, Ivan V. Kulakov

**Affiliations:** 1 The Faculty of Veterinary Medicine, Omsk State Agrarian University Named after P.A. Stolypin, 2 Institutskaya Ploshchad, 644008 Omsk, Russia; 2 Omsk Agrarian Scientific Center, 26 Koroleva Ave., 644012 Omsk, Russia; 3 Center of Nature-Inspired Engineering, University of Tyumen, 15a Perekopskaya St., 625003 Tyumen, Russia

**Keywords:** guinea pigs, tuberculosis, BCG vaccine, betulin, betulinic acid, betulonic acid, BALT

## Abstract

Tuberculosis caused by *Mycobacterium bovis* is a serious problem for animal and human health worldwide. A promising concept for the design of anti-tuberculosis drugs is the conjugation of an immunogenic fraction isolated from bacterial vaccines with a stimulating component. Taking this principle as a basis, conjugates based on BCG antigens with betulin and its derivatives (betulonic and betulinic acids) were designed. The aim of this research was to study the morphological changes in the lymphoid tissue associated with the bronchial mucosa lungs (BALT) in guinea pigs sensitized with experimental conjugates using a model of experimental tuberculosis. The results showed a significant decrease in the BALT response, expressed by a decrease in the diameter of lymphatic follicles and a decrease in their activity when exposed to conjugates based on BCG antigens with betulin and, especially, with betulonic acid, with a visually greater number of plasma cells observed in the lung tissues of guinea pigs of these groups. The absence of tuberculous foci and low BALT activity in the lungs of animals treated with betulin and betulonic acid are probably associated with the activation of humoral immunity under the action of these conjugates.

## 1. Introduction

Tuberculosis (TB) is a known worldwide infection that is constantly on the list of the most-deadly diseases [1]. Back in the 18th and 19th centuries, it caused waves of epidemics that raged in European countries, which were replaced by a period of great inventions of the 20th century for global health, such as purified protein derivative (PPD) tests and Bacille Calmette–Guérin (BCG) vaccine [2]. However, despite the fact that humanity has long been familiar with TB and developed numerous treatment approaches, this infection still cannot be completely defeated.

The causative agent of TB in humans and some mammals is usually *Mycobacterium tuberculosis* (*Mtb*) or Koch’s tubercle bacillus named after its investigator [2]. Since the discovery of *Mtb*, a large number of mutations in bacillus genome have occurred that allowed this pathogen to be resistant to once-effective drugs and cause damage to human health [3]. Particularly the problem of drug resistance has become especially acute during the COVID-19 pandemic when 1.3 million deaths from TB were registered only in 2020, according to the WHO Global TB Report [1]. *Mycobacterium bovis* (*M. bovis*) can be distinguished as another significant representative of the *Mycobacterium tuberculosis* complex (MBTC) [4,5,6]. The characteristic host for these species is cattle, but it does not eliminate the ability to infect other mammals, including humans [5,6]. *M. bovis* is also considered the parent of the *M. bovis* BCG strain, which is the main component of the BCG vaccine [7]. The end symptoms for the infected may differ in the case of human or zoonotic TB [4]; therefore, it is important to identify the nature of the pathogen and establish the mechanism of its action in the early stages of the disease.

As an example of a possible indicator of tuberculosis infection spreading, primarily in animals, the formation of bronchus-associated lymphoid tissue (BALT) may be highlighted [8]. For the first time, lymphoid tissue coupled with the bronchial mucosa was found in pigs and rabbits and appeared as an accumulation of lymphoid cells directly under the epithelium [9,10]. In recent years, BALT and its role in the formation and maintenance of immunity under the action of various pathogenic factors, including mycobacteria, has attracted considerable attention from scientists. Moreover, the role of BALT is similar to the role of Peyer’s patches of the small intestine and other non-encapsulated lymphoid formations in the form of follicles. According to a number of authors, it is involved in antigen recognition, initiation of an immune response, and dissemination of primary lymphoid cells in the respiratory tract [9,11].

It is worth mentioning that BALT is not a permanent structure in all animals, i.e., its occurrence in the lungs varies widely. It is found in the lungs of rabbits in 100% of cases, in guinea pigs in 50%, and in pigs in 33%. In cats and humans, it is completely absent in unchanged lungs [10], but it can be formed after antigenic stimulation [12,13].

Another distinguishing feature of the BALT is the formation of a response to antigen exposure, which is called inducible bronchus-associated lymphoid tissue (iBALT) [9]. They are similar structurally and functionally; therefore, both abbreviations can be considered interchangeable. Stationary BALT lymphoid formations are located near the bifurcation of large bronchi, usually between the artery and the bronchus and under the bronchial epithelium. Lymphoid tissue is also found near blood vessels and in the interstitium of the lower sections of the lungs [13].

A number of studies have shown that the formation of BALT is also associated with *Mtb* infection, which causes pulmonary tuberculosis. During the course of the disease, there is a temporary “paralysis” of the migration of dendritic cells into the lymph nodes that drain the lungs [14,15], where the delay of generation of the Th1 and Th17 responses occurs [16,17], which leads to the accumulation of mycobacteria in infected macrophages [17,18,19]. Even when the Th1 and Th17 response occurs, *Mtb* survives but resides in granulomas, an inducible lymphoid structure with a central zone of infected macrophages, which are surrounded by activated T and B cells [20]. These activated B and T cells often form iBALT around granulomas in infected humans and animals [20,21,22].

Moreover, active stimulation of BALT can occur both in mice and in guinea pigs after the administration of the BCG vaccine [23,24]. This explains the very early lymphocytic response seen in vaccinated animals. The analysis of the literature showed that studies concerning the morphological changes in the lymphoid tissue of the guinea pigs’ lungs experimentally infected with mycobacteria and vaccinated with BCG are poorly reflected in scientific publications. The available reports on the effect of immunogens from individual fractions of the destroyed BCG culture conjugated with an immunostimulating matrix are mainly devoted to the study of their effect on the immune system [25], while morphological changes are described only in a few works.

**Chart 1:**
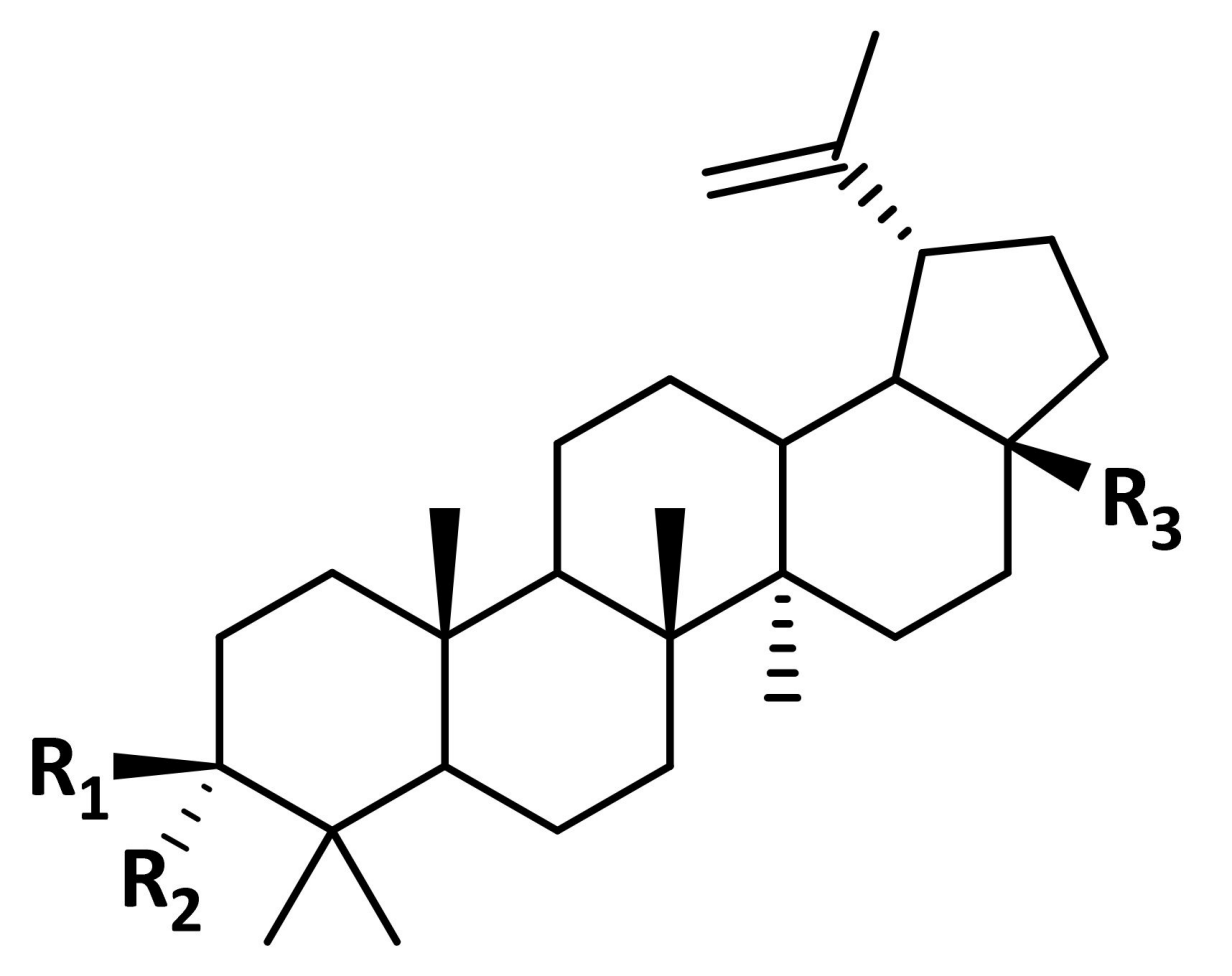
Chemical structure of betulin (R_1_ = OH, R_2_ = H, R_3_ = CH_2_OH), betulonic (R_1_ + R_2_ = O, R_3_ = COOH), and betulonic (R_1_ = OH, R_2_ = H, R_3_ = COOH) acids.

Bringing all mentioned above together, the purpose of this research was to study, using histological and morphometric methods, the morphological changes in the lymphoid tissue of the lungs (BALT) of guinea pigs immunized with BCG vaccine when infected with a culture of *M. bovis* and in the presence of betulin, betulonic, and betulinic acids (Chart 1).

## 2. Materials and Methods

### 2.1. Pharmacophore Models and Molecular Docking

The 3D structures of betulin, betulonic, and betulinic acids (Chart 1) were constructed based on the crystal structure of betulinic acid (CCDC Deposition Number 829776), and their geometry was further optimized using the Avogadro software package [26]. Crystal structures of applied proteins were extracted from the Protein Data Bank (PDB) [27]. All proteins and small molecules were prepared and reformatted in pdbqt files using BIOVIA Discovery Studio 2020 [28] and AutoDock tools [29]. Molecular docking was performed using AutoDock Vina 1.2.3 with vina scoring function and grid size 18 × 18 ×18 Å^3^ for all studies [30,31]. Pharmacophore models, as well as obtained conformations of small molecules and their interactions with proteins, were investigated in BIOVIA Discovery Studio 2020 [28].

### 2.2. In Vivo Anti-Tuberculosis Activity Study

Experimental studies were performed with male agouti guinea pigs aged 4–5 months with a mass of 400–450 g. The animals were kept in a specialized vivarium of the epizootiology laboratory and tuberculosis control measures of the Omsk Agrarian Scientific Center veterinary department.

For the formation of conjugates based on BCG antigens, betulin, betulonic, and betulinic acids (Chart 1) were used, the preparation of which was carried out in accordance with previously proposed methods [32,33,34].

Five groups of five guinea pigs were formed for the experiment. The animals of group one received subcutaneous injections of physiological saline (0.5 mL). Guinea pigs of groups two, three, and four received a subcutaneous injection of prepared complex of BCG antigens with betulinic acid, betulin, and betulonic acid, respectively (0.5 mL, protein concentration 1 mg/mL). Group five was intradermally immunized with the BCG vaccine (0.1 mg in 0.1 mL of saline). Drug injection was carried out only once since the double and triple administration of inoculation in short intervals between injections reduces the level of immune protection of laboratory animals.

On the 30th day after inoculation, guinea pigs from all groups were infected with a virulent culture of *M. bovis* (strain 14), which was injected subcutaneously at a dose of 0.001 mg/mL. Forty-five days after the experimental infection, the animals were subjected to diagnostic euthanasia.

To obtain antigenic complexes, the culture of the BCG vaccine strain grown in Sauton’s fluid medium base was subjected to ultrasonic disintegration by a UZDN-1 device (22–35 kHz, 60–70 W/cm^2^) for 30 min.

For histological studies, lung pieces with a size of 1.5 × 1.5 cm^2^ from areas without visible specific changes were fixed in 10% neutral formalin. Then, they were placed in paraffin with preliminary dehydration and compaction in a number of alcohol solutions of ascending concentration. Tissue slices with thicknesses from 7 to 10 μm were stained with hematoxylin and eosin, after which they were embedded in Canada balsam.

For the morphometry of BALT lymphatic follicles, a Zeizz AXIO Imager A1 binocular microscope, digital camera, and the ScopePhoto program were used. A built-in ocular reticle was used to determine the diameter (D), radius (R), and area (S) of the follicles. These parameters were measured for five follicles of each guinea pig.

The obtained digital material was presented in the form of the mean (M) and standard error of the mean (m) in accordance with the Student *t*-values. The results were considered reliable at *p* ≤ 0.05.

## 3. Results and Discussion

In the first instance, we searched the DrugBank database [35] for records of drugs that improve the effectiveness of the BCG vaccine. In the list of 496 entries with the marker “Approved”, 16 drugs were discovered that enhance the therapeutic efficacy of the BCG vaccine and have a gonane fragment in their structure. Notably, no triterpenoids or hopane-related compounds were found on the list. For all selected drugs, the Tanimoto coefficients [36] were calculated using ChemMine Tools [37] (Table 1). This index is usually used to evaluate the degree of the chemical similarity of two compared structures.

The lowest and the largest Tanimoto coefficients were noticed for pairs Deflazacort–betulonic acid (0.3540) and Hydrocortisone–betulonic acid (0.5128), respectively (Table 1). These values are low, which indicates poor structural similarity and, therefore, the low probability of exhibiting identical biological properties of selected steroids and betulin derivatives. On the other hand, all selected from the list of steroid-like drugs also have a wide range of Tanimoto coefficients; however, indeed, many of them improve the effectiveness of the BCG vaccine and the resistance to the spread of TB infection [38,39,40,41,42,43]. For instance, the Tanimoto coefficient for Budesonide and Flunisolide is 0.7879, and they both demonstrated anty-cytolysis activity against *Mtb* [42], while the Tanimoto coefficient for Budesonide and Trilostane is 0.4474, which does not prevent the latter from inhibiting the functions of *Mtb* proteins [43]. Moreover, it is known that in some cases, hopanoids are able to replace sterols, for example, in the construction of cell membranes [44]. These observations motivate further analysis of the structure of betulin derivatives and their interaction with target proteins.

**Figure 1:**
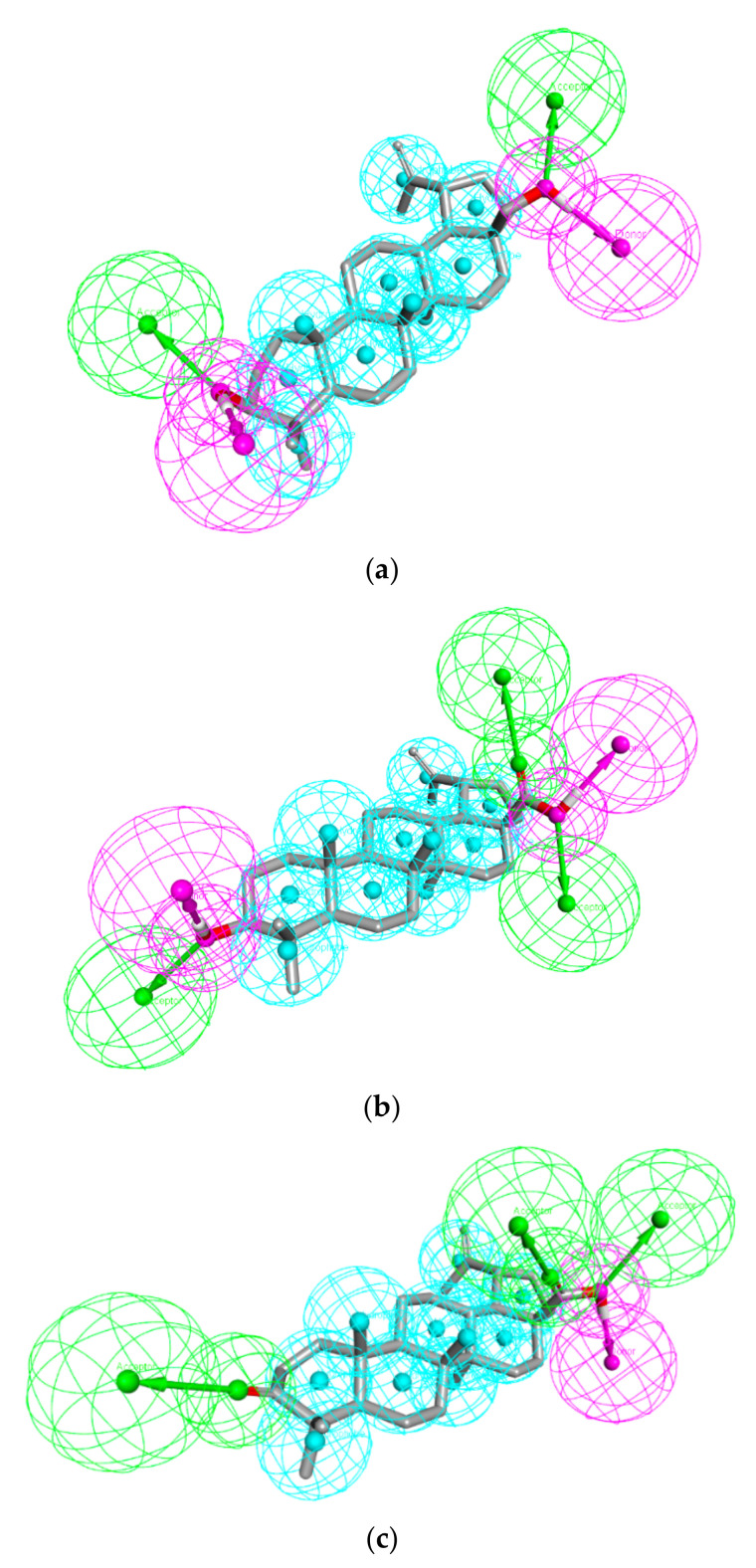
Pharmacophore models for (**a**) betulin, (**b**) betulinic, and (**c**) betulonic acids. Color code: cyan = hydrophobic; green = H-acceptor; purple = H-donor.

Betulin and its close derivatives, betulonic and betulinic acids, are more than 90% structurally similar. Their common hopane motif is responsible for hydrophobic interactions with a potential protein target (Figure 1). As for the centers involved in the formation of intermolecular hydrogen bonds, their largest number is observed in betulinic acid (Figure 1). The predominant amount of hydrogen acceptor centers is observed in betulonic acid, in which the carbonyl group at the 3-position additionally fixes the geometry of the adjacent cyclohexyl ring and accordingly reduces the number of possible energetically favorable conformations in comparison with the other two derivatives (Figure 1). From this point of view, betulinic acid has greater conformational capabilities for the formation of stable complexes with biological macromolecules.

**Figure 2:**
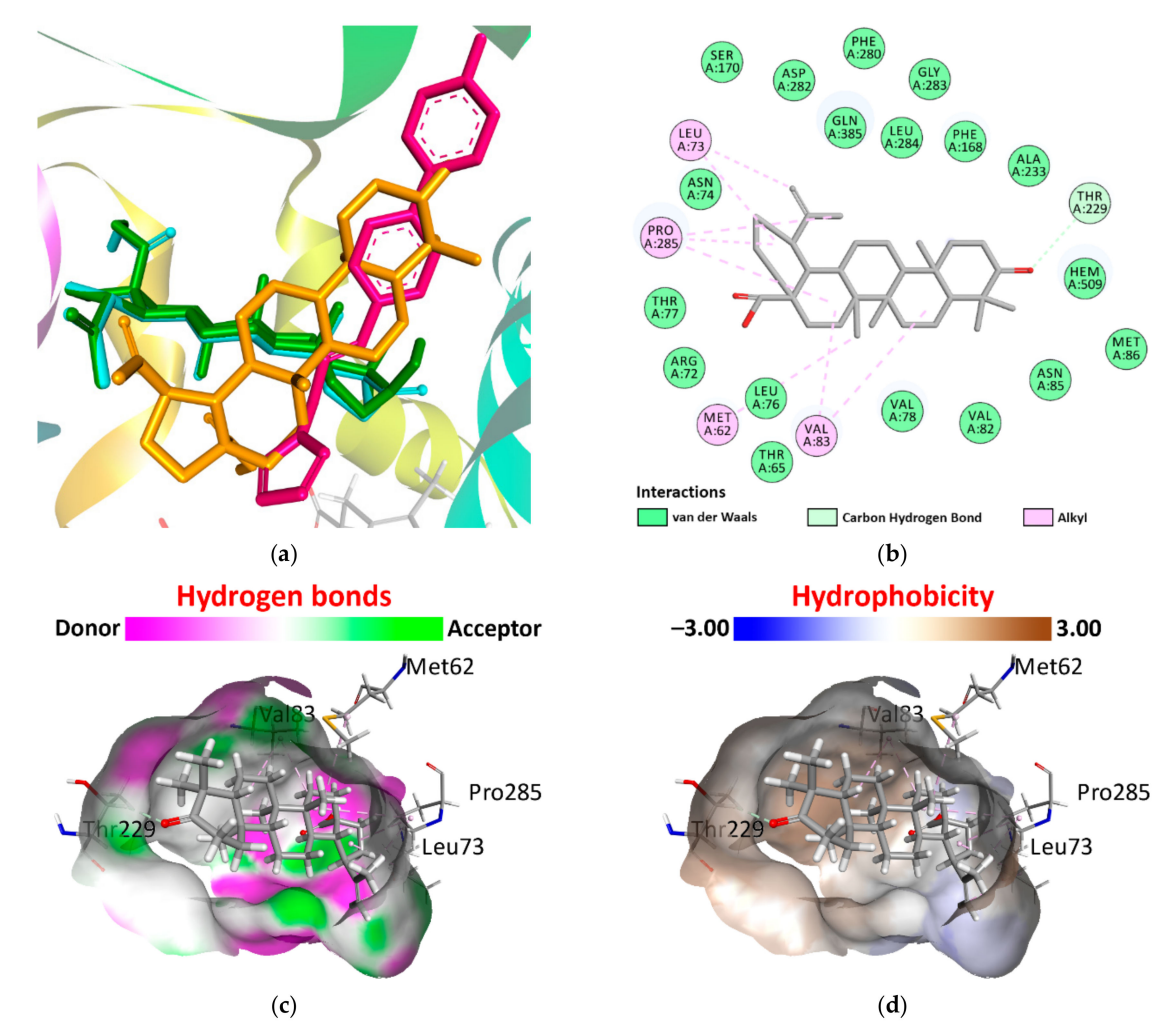
(**a**) Docking poses for N5Z (pink), betulin (green), betulonic (cyan), and betulinic (orange) acids in active site of *Mtb* CYP121 protein; (**b**) 2D plot of interactions between *Mtb* CYP121 and betulonic acid; (**c**) hydrogen bonds and (**d**) hydrophobicity surfaces of *Mtb* CYP121 active site.

After building pharmacophore models, we performed molecular docking with protein targets that may be structural parts of the *Mtb* and/or *M. bovis* (Table 2). It was noticed that binding energy decreases slightly in the series betulinic acid–betulin–betulonic acid for carbohydrate-recognition domain of the C-type lectin mincle and *Mtb* CYP121, whereas complexes with *Mtb* adenosine kinase and UDP-*N*-acetylmuramic Acid L-alanine ligase (MurC) are characterized by an energetic decrease in the series betulin–betulinic acid–betulonic acid (Table 2). Additionally, the most efficient protein–ligand binding, compared with the calculated pK*_i_* values for the initial ligands, was observed for complexes of Carbohydrate-recognition domain of the C-type lectin mincle with all the studied compounds and complex of *Mtb* CYP121 with betulonic acid (Table 2). For the last complex, the lowest binding energy among all complexes was found, and it equals −11.02 kcal/mol (Table 2). The location of betulonic acid in the active site of *Mtb* CYP121 does not match the location of the original ligand, but it does coincide with betulin (Figure 2). However, the corresponding pK*_i_* values for betulin and betulonic acid differ significantly (Table 2). The resulting interaction of betulonic acid with *Mtb* CYP121 active site includes one C···H bond with Thr229 and eight alkyl···alkyl interactions with Met62, Leu73, Val83, and Pro285 and is supported by van der Waals forces (Figure 2). Moreover, betulonic acid is predominantly located along the hydrophobic surface of *Mtb* CYP121 active site, which also favorably stabilizes this complex (Figure 2). Thus, betulonic acid is distinguished from the other two derivatives by its pronounced activity in relation to a series of selected proteins.

**Figure 3:**
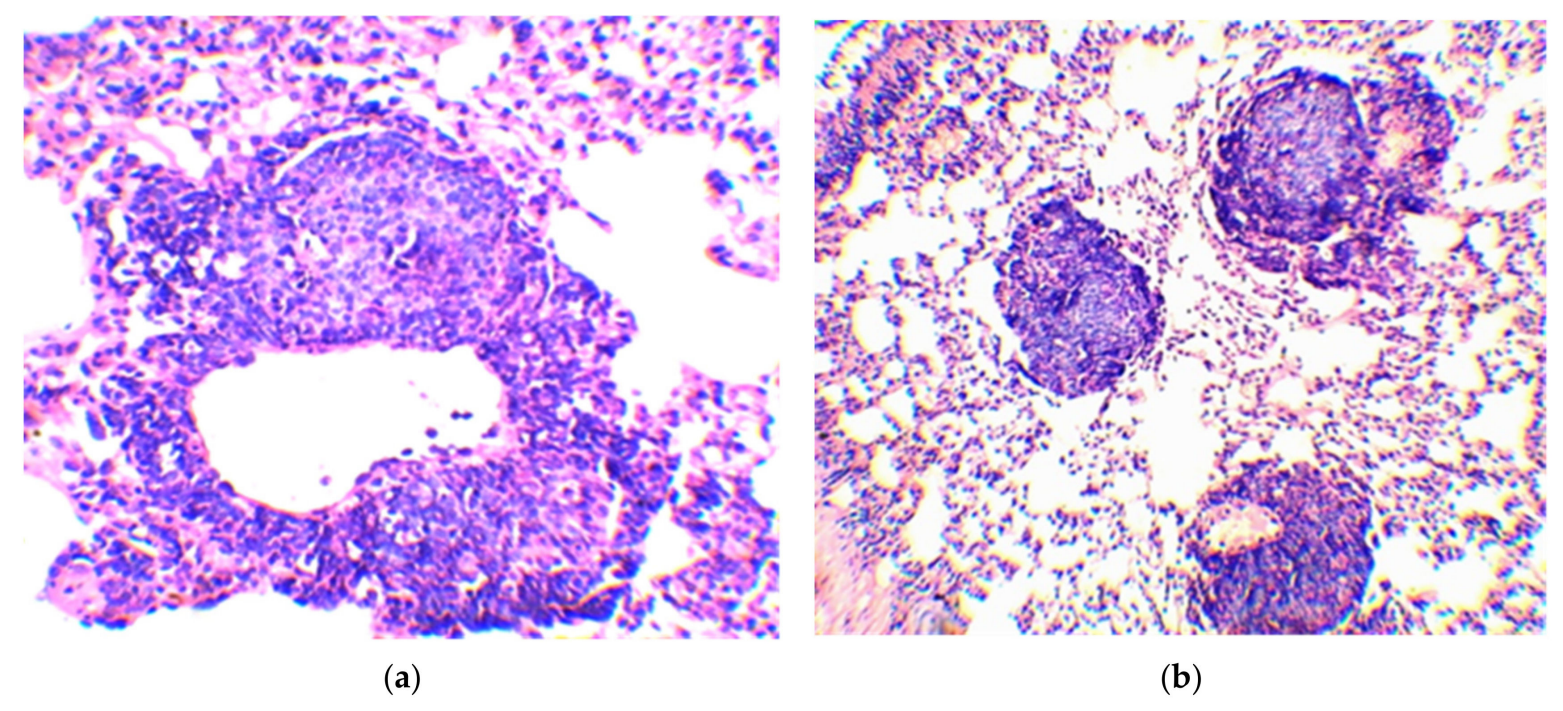
Guinea pig lung from group one. (**a**) Two lymphatic follicles with reactive centers forming near the wall of the vessel (magnification of 100×). (**b**) Lymphatic follicles with a pronounced reproduction center. A connection with a blood vessel is observed in the lower follicle (magnification of 5×).

After preliminary computational assays, we performed in vivo experiments with guinea pigs. An accumulation of lymphoid cells was observed almost around all blood vessels of the lung parenchyma of guinea pigs in group one, in some places forming a dense cellular accumulation (Figure S1–S10). The width and length of observed cell couplings differ around the circumference of the vessel. In some cellular accumulations, the proliferation of lymphoid tissue occurs eccentrically with the formation of a typical follicle (Figure 3). Moreover, the centers of reproduction (germinal centers), which are formed due to the multiplication of reticulocytes and macrophages, appear at the core of many emerging follicles (Figure 3). On a series of sections, the connection of most of the lymphatic follicles with blood vessels can be traced. In addition, there are fewer accumulations of lymphoid tissue near the bronchi than around the vessels.

The morphometric studies have shown that the lymphatic follicles of infected guinea pigs from group one have a diameter of 203.0 ± 8.8 µm and an area of 34,103.0 ± 2766.5 µm^2^ (Table 3). At the same time, most of the lymphatic follicles (40%) have a diameter from 170 to 220 µm (Table 3). Follicles with diameters from 230 to 270 µm (26.6%) and from 110 to 160 μm (23.3%) take the second and third places, respectively, in terms of quantity (Table 3).

**Figure 4:**
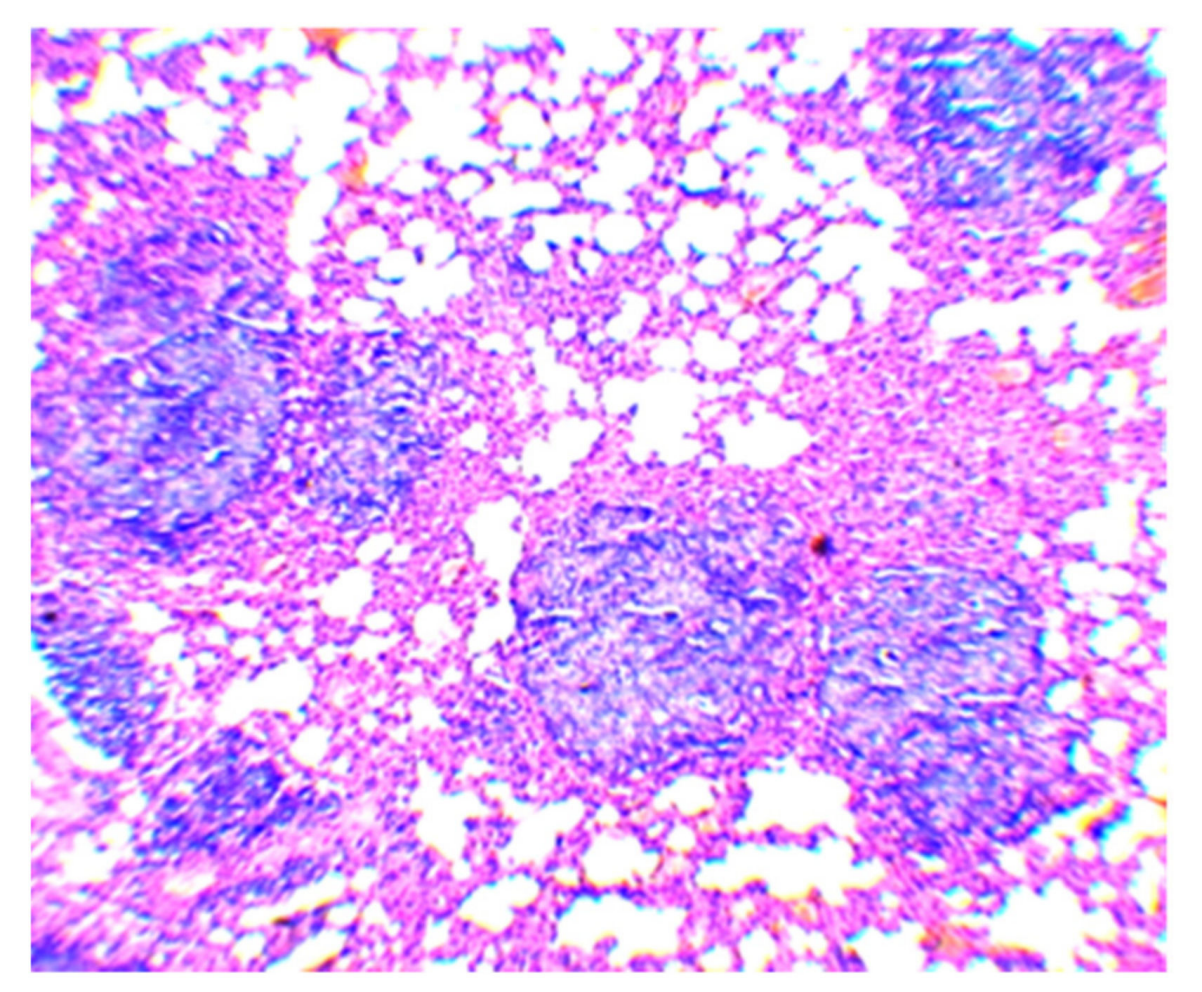
Guinea pig lung immunized with BCG antigens and betulinic acid. A large number of lymphatic follicles with generative centers in the lung tissue is observed. The centers of reproduction are expressed in the follicles (magnification of 50×).

The second group of guinea pigs treated with a complex of BCG antigens and betulinic acid demonstrated the formation of a large number of lymphatic follicles, in some of which the reproduction zone was well expressed (Figure 4, Figures S11–S17). The corresponding mean area and diameter for group two are 37,478.8 ± 4532.1 µm^2^ and 216.3 ± 14.0 µm, respectively (Table 3). As in the case of group one, in group two, the most common follicle diameter was from 170 to 220 µm (33.3%) (Table 3). A slightly smaller number of follicles (25.7%) had a diameter from 230 to 270 µm, whereas follicles with a diameter from 110 to 160 µm and from 280 to 380 µm each separately make up 16.7% (Table 3).

We noted that the proliferation of lymphocytes around the blood vessels and bronchi with the formation of cell couplings in guinea pigs of group three, which received a complex of BCG antigens with betulin (Figures S18–S22), is much less pronounced than in guinea pigs from previous groups. As in guinea pigs from groups one and two, lymphatic follicles were found in the lungs, but their number and size are visually smaller than in the mentioned groups. The reproduction zone in most follicles is not expressed. Unlike the guinea pigs of group one, in the lungs of the guinea pigs of group three, as well as in group two, a large number of plasma cells were found among the macrophages proliferating between the alveoli. During morphometry, it was observed that the average diameter (147.0 ± 9.0 µm) and area (19,080.7 ± 1905.0 µm^2^) of follicles in the lungs of guinea pigs from group three take on much smaller values comparing parameters of infected, but not vaccinated, guinea pigs (Table 3). The largest number (63.3%) of observed follicles have a diameter from 70 to 160 µm (Table 3).

In the lungs of guinea pigs from group four, which received a complex of BCG antigens with betulonic acid (Figures S23–S27), a large number of plasma cells is found among the cells proliferating in the interalveolar tissue. The proliferation of lymphocytes around the blood vessels and bronchi with the formation of cellular accumulation is much less pronounced than in guinea pigs of the first and second groups. Near the walls of bronchi of different caliber, the aggregation of lymphoid cells is either absent or insignificant. Lymphoid cell couplings are also absent around most blood vessels, and if they are present, they are also small in width. The formation of follicles in the perivascular couplings is not observed. The number of lymphatic follicles in the lungs of guinea pigs from group four is visually less than in other groups. The reproduction zone is not expressed in the follicles. Additionally, the smaller size of lymphatic follicles is also confirmed by morphometric data. The average diameter and area of follicles in the lungs of animals from group four are equal to 115.7 ± 6.5 μm and 11,458.4 ± 1240.7 μm^2^, respectively (Table 3).

**Figure 5:**
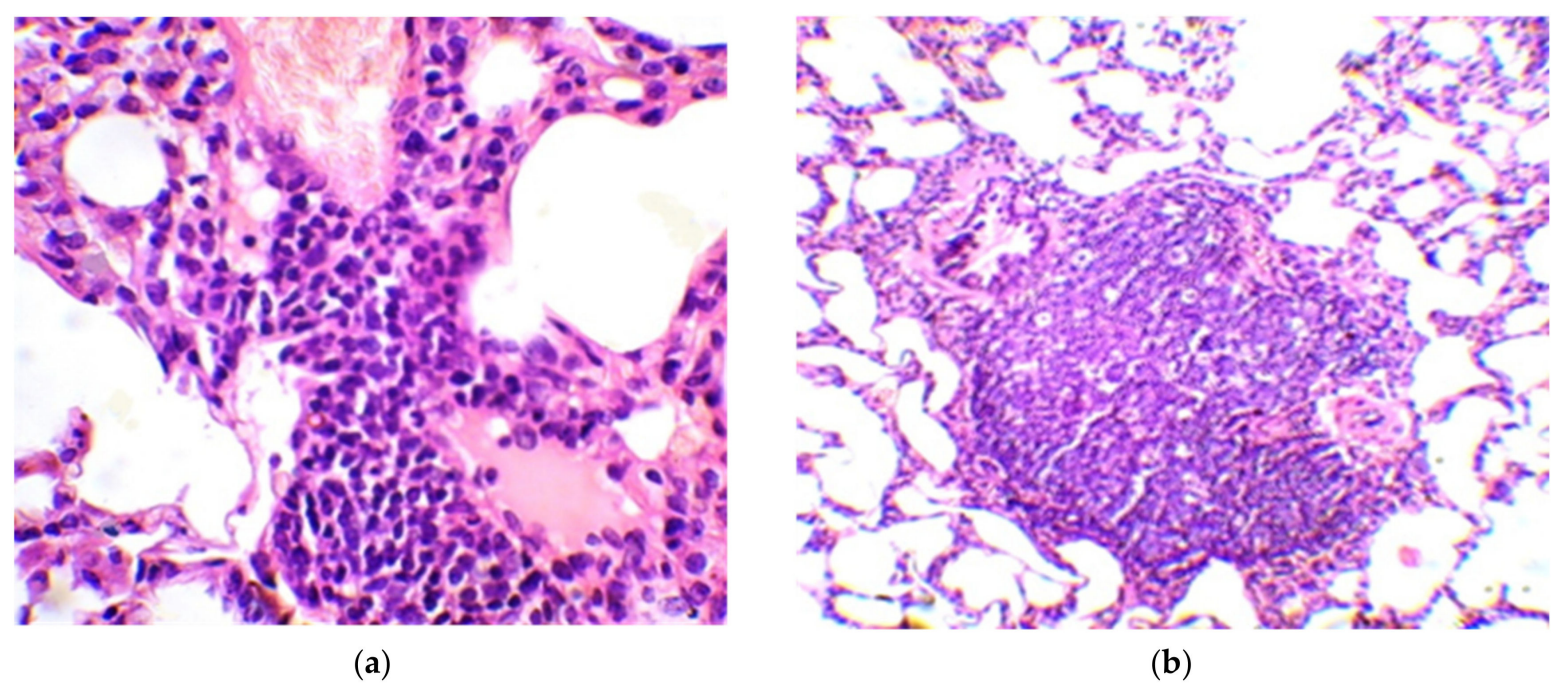
Lung of a guinea pig immunized with BCG vaccine. (**a**) There are few perivascular couplings that show no signs of lymphatic follicle formation (magnification of 200×). (**b**) There is a large lymphatic follicle located between the bronchus and artery (magnification of 50×).

In guinea pigs from group five, which were vaccinated intradermally with the BCG vaccine (Figures S28–S32), in contrast to infected unvaccinated guinea pigs, the proliferation of epithelioid macrophages in the interalveolar tissue is less pronounced. In vaccinated animals, there are significantly fewer clusters near the bronchi, blood vessels, and follicles in the lymphoid tissue. Lymphoid cells form small clusters along the bronchi. Perivascular couplings made of lymphoid cells have a small width and length (Figure 5). A pronounced process of formation of lymphatic follicles in perivascular couplings is not observed. Meanwhile, in the formed follicles, the connection with the blood vessels is maintained (Figure 5). In contrast to the guinea pigs of group one, the generative and reactive centers are not expressed in the lymphatic follicles. The ratio of follicles by size also significantly differs from other groups. Follicles with a diameter from 70 to 100 μm in vaccinated animals make up 13.3%, while the number of follicles with a diameter from 110 to 160 μm is almost twice as much as in guinea pigs of group one (50.0% and 23.3%, respectively) (Table 3). Moreover, the number of follicles with a diameter from 170 to 220 μm decreases compared with group one (40.0%) and equals 23.4% (Table 3). The differences in the content of follicles with a diameter of 230–270 μm in unvaccinated and intradermally vaccinated guinea pigs are also pronounced (26.7% and 13.3%, respectively), while the largest diameter in the group of vaccinated animals was 250 μm (Table 3). In the unvaccinated animals, the number of follicles with a diameter of 280–300 μm is 6.7%, and in the vaccinated, they were not found (Table 3).

During a histological examination of the lungs of guinea pigs that were parenterally infected with *M. bovis* and subjected to euthanasia after 45 days, it was established that by this period, an immune response to the generalization of the pathogen occurs in the organ, which consists in the proliferation of lymphocytes around blood vessels and bronchi of different caliber, and the formation of typical lymphatic follicles. At the same time, lymphoid tissue in the form of cell couplings of various widths and lengths was found in a greater amount around the blood vessels, which indicates hematogenous dissemination of the pathogen. Growing eccentrically, the lymphoid tissue of the perivascular couplings forms follicles, in the core of which centers of reproduction and reactive centers were formed. Due to the fact that in the lungs of infected guinea pigs, there are a large number of follicles with centers of reproduction and reactive centers, we believe that the lymphoid formations present in the organ relate to induced BALT, which is evidence of antigenic stimulation caused by the presence of the pathogen in the blood. According to a number of authors, induced BALT is formed in the lungs in response to antigen exposure and includes a set of lymphoid follicles with a pronounced germinal center, a large number of B cells surrounded by a zone of T cells [10,12,13].

The absence of tubercles with an active process in the lungs of infected guinea pigs is possibly the result of the action of activated BALT. The possibility of the impact of the BALT system on the course of the tuberculosis process is indicated in the studies of lung morphology during infection with *Mtb* [20,22].

In guinea pigs, which were vaccinated intradermally with the BCG vaccine before infection with the culture of *M. bovis*, and euthanized 45 days after infection, a significantly lower amount of lymphoid tissue around the vessels and bronchi was observed than in unvaccinated animals. The diameter of the follicles was also significantly smaller. In these follicles, the centers of reproduction were not clearly expressed, and the reactive centers were not found. In our opinion, this is due to a decrease in the concentration of antigens in the blood as a result of the development of immunity. A number of studies also indicate the development of immunity in the lungs during BCG vaccination, leading to a decrease in the growth of mycobacteria [45]. The fact that BCG vaccination can influence the formation of granulomas in the lungs upon infection with *Mtb* is indicated in the work of Grover A. et al. [46]. On day 15, after aerogenic infection of guinea pigs with *Mtb*, the authors observed that in unvaccinated guinea pigs, individual cell clusters scattered throughout the lung in the perivascular zones. They had an irregular shape and consisted mainly of mononuclear cells. In vaccinated animals, the foci were also perivascular, there were more of them, and they were clearly demarcated from the surrounding tissue. In the center of the granulomas, there were macrophages. There was a ring of lymphocytes around them. In our experiment, during the parenteral infection of guinea pigs with *M. bovis*, a different morphological reaction was observed on the 45th day.

## 4. Conclusions

The primary computational analysis of betulin, betulonic, and betulinic acids led to the conclusion that these compounds have poor structural similarity with a series of sterols, which improve the therapeutic effect of the BCG vaccine. However, as a result of molecular docking, betulonic acid, in most cases, showed the highest inhibitory activity against protein targets that may be structural parts of the *Mtb* and/or *M. bovis*.

During the study of the possible effect of betulin and its derivatives on the morphology of the tuberculosis process in the lungs of guinea pigs, we noted a significant decrease in the BALT reaction, which was primarily expressed in a reduction of the diameter of lymphatic follicles and decrease in their activity under the influence of betulin and, especially, betulonic acid. At the same time, no significant differences in morphological and morphometric parameters were observed in animals sensitized by the conjugate of BCG antigens with betulinic acid and infected but not vaccinated animals. Moreover, in the lung tissue of guinea pigs treated with conjugates of BCG antigens with betulin and betulonic acid, a greater number of plasma cells was visually observed. This allows us to assume that the absence of these tuberculous foci groups in the lungs of animals and the low activity of BALT are associated with the activation of humoral immunity under the action of betulin and its derivatives.

## Figures and Tables

**Table 1:** Results of maximum common substructures (MCS) search for betulin, betulonic, and betulinic acids.

Drug Name	MCS Tanimoto Coefficient ^1^		
	Betulin	Betulonic Acid	Betulinic Acid
Betamethasone	0.4286	0.4186	0.4186
Budesonide	0.4000	0.3617	0.3913
Cortisone acetate	0.4524	0.4762	0.4419
Deflazacort	0.3913	0.3540	0.3830
Dexamethasone	0.4286	0.4186	0.4186
Fludrocortisone	0.4750	0.5000	0.4634
Flunisolide	0.4000	0.3617	0.3913
Fluprednisolone	0.4390	0.3953	0.4286
Fusidic acid	0.4082	0.3725	0.4000
Hydrocortisone	0.4873	0.5128	0.4750
Meprednisone	0.4390	0.4286	0.4286
Methylprednisolone	0.4390	0.4286	0.4286
Prednisolone	0.4500	0.4048	0.4390
Prednisone	0.4500	0.4048	0.4390
Triamcinolone	0.4286	0.3864	0.4186
Trilostane	0.4737	0.4615	0.4615

^1^ All coefficients were obtained using ChemMine Tools [37].

**Table 2:** Molecular docking results for complexes of listed proteins with their initial ligands ^1^, betulin, betulonic, and betulinic acids.

Protein	PDB ID	Ligand	Binding Energy (kcal/mol)	pK*_i_*
Carbohydrate-recognition domain of the C-type lectin mincle	4KZW	FLC	−4.20	3.06
		Betulin	−5.97	4.35
		Betulonic acid	−6.02	4.39
		Betulinic acid	−5.81	4.23
*Mtb* adenosine kinase	4UBE	2FA	−9.92	7.23
		Betulin	−6.84	4.98
		Betulonic acid	−7.01	5.11
		Betulinic acid	−6.92	5.04
*Mtb* CYP121	6TET	N5Z	−9.80	7.14
		Betulin	−9.79	7.13
		Betulonic acid	−11.02	8.03
		Betulinic acid	−9.52	6.94
UDP-*N*-acetylmuramic Acid L-alanine ligase (MurC)	7BVB	UD1	−7.94	5.78
		Betulin	−7.45	5.43
		Betulonic acid	−7.66	5.58
		Betulinic acid	−7.53	5.49
*Mtb* tryptophanyl-tRNA synthetase	7ENS	ATP	−8.92	6.50
		5BX	−7.95	5.79
		Betulin	−5.39	3.93
		Betulonic acid	−6.24	4.55
		Betulinic acid	−7.12	5.19

^1^ FLC = citrate anion; 2FA = 2-fluoroadenosine; N5Z = 1-[(~{E})-3-[4-(4-fluorophenyl)phenyl]prop-2-enyl]imidazole; UD1 = uridine-diphosphate-*N*-acetylglucosamine; 5BX = (5*S*)-5-[(1*R*)-1-(1*H*-indol-3-yl)ethyl]-2-(methylamino)-1,3-oxazol-4(5*H*)-one.

**Table 3:** Morphometric parameters of BALT lymphatic follicles in guinea pigs infected with *M. bovis*.

Group No.	BCG Vaccine	Compound	Mean Follicle Area (µm^2^)	Mean Follicle Diameter (µm)	Follicle Size Distribution by Diameter (µm)	Number of Follicles of the Same Size (%)
1	−	−	34,103.0 ± 2766.5	203.0 ± 8.8	70–100	3.3
					110–160	23.3
					170–220	40.0
					230–270	26.7
					280–300	6.7
2	+	Betulinic acid	37,478.8 ± 4532.1	216.3 ± 14.0	70–100	6.7
					110–160	16.7
					170–220	33.3
					230–270	25.7
					280–300	16.7
3	+	Betulin	19,080.7 ± 1905.0 ^1^	147.0 ± 9.0 ^2^	70–100	10.0
					110–160	53.5
					170–220	36.7
4	+	Betulonic acid	11,458.4 ± 1240.7 ^2^	115.7 ± 6.5 ^2^	70–100	43.3
					110–160	46.7
					170–190	10.0
5	+	−	20,742.3 ± 2386.4 ^1^	155.0 ± 9.1 ^2^	70–100	13.3
					110–160	50.0
					170–220	23.4
					230–250	13.3

^1^ Result is obtained with *p* < 0.01. ^2^ Result is obtained with *p* < 0.001.

## Data Availability

Not applicable.

## Supplementary Materials

The following supporting information can be downloaded at: https://www.mdpi.com/article/10.3390/vaccines10122084/s1, Figure S1: A tuberculous granuloma in contact with the wall of a blood vessel for group one (not vaccinated); Figure S2: Accumulation of lymphoid tissue near the bronchus for group one (not vaccinated); Figure S3: Lymph nodule between bronchi and associated vessels for group one (not vaccinated); Figure S4: A lymph node inculcation in lymphatic vessel for group one (not vaccinated); Figure S5: Couplings of lymphoid cells around hyperemic vessels for group one (not vaccinated); Figure S6: Cellular couplings on a transverse section of the connecting vessel for group one (not vaccinated); Figure S7: Eccentric growth of the lymphoid cells of the coupling, followed by the formation of a follicle for group one (not vaccinated); Figure S8: Two lymphatic follicles with reactive centers forming near the vessel wall for group one (not vaccinated); Figure S9: Lymph follicles of varying sizes with eccentric blood vessels (right) for group one (not vaccinated); Figure S10: Lymphatic follicles with a pronounced reproduction center for group one (not vaccinated); Figure S11: Compacted area under the pleura as a result of proliferation of epithelioid macrophages for group two (vaccinated with BCG + betulinic acid); Figure S12: A site compacted as a result of proliferation in the walls of the alveoli of epithelioid cells for group two (vaccinated with BCG + betulinic acid); Figure S13: Lymphatic follicle near the branches of the large bronchi for group two (vaccinated with BCG + betulinic acid); Figure S14: A coupling with a small number of lymphocytes around the blood vessel, among the thickened macrophages as a result of proliferation in the walls of the alveoli of epithelioid for group two (vaccinated with BCG + betulinic acid); Figure S15: A small number of lymphocytes around the blood vessel, which is among the overgrown epithelioid macrophages for group two (vaccinated with BCG + betulinic acid); Figure S16: Among epithelioid cells, there are a large number of cells with bright red cytoplasm–plasmocytes for group two (vaccinated with BCG + betulinic acid); Figure S17: A large number of lymphatic follicles with generative centers in the lung tissue for group two (vaccinated with BCG + betulinic acid); Figure S18: Lymphatic follicle at the branching site of medium-sized bronchi for group three (vaccinated with BCG + betulin); Figure S19: A blood vessel in the area of the lungs compacted as a result of the proliferation of epithelioid macrophages for group three (vaccinated with BCG + betulin); Figure S20: The initial stage of formation of a lymphatic follicle near a blood vessel for group three (vaccinated with BCG + betulin); Figure S21: One of the few follicles with a breeding center for group three (vaccinated with BCG + betulin); Figure S22: Plasma cells (cells with bright pink cytoplasm) among epithelioid macrophages, as well as near a small lymphatic follicle for group three (vaccinated with BCG + betulin); Figure S23: Areas of lung tissue compaction of various sizes for group four (vaccinated with BCG + betulonic acid); Figure S24: A small accumulation of lymphoid cells near a medium-sized bronchus for group four (vaccinated with BCG + betulonic acid); Figure S25: Cellular couplings of a small number of lymphocytes around a blood vessel in the area of proliferation of epithelioid macrophages for group four (vaccinated with BCG + betulonic acid); Figure S26: Plasma cells (cells with bright pink cytoplasm) in the walls of the alveoli for group four (vaccinated with BCG + betulonic acid); Figure S27: The small size follicle without a pronounced reproduction center for group four (vaccinated with BCG + betulonic acid); Figure S28: Accumulation of lymphoid cells near the bronchus and artery for group five (vaccinated with BCG); Figure S29: A small collection of lymphoid cells in the form of a nodule in the peribronchial tissue for group five (vaccinated with BCG); Figure S30: Lymph follicles maintain contact with blood vessels for group five (vaccinated with BCG); Figure S31: A large lymphatic follicle located between the bronchus and the artery for group five (vaccinated with BCG); Figure S32: A few perivascular couplings show no evidence of lymphatic follicle formation for group five (vaccinated with BCG).
