# Supplementary material for: Morphology of Lymphoid Tissue in the Lungs of Guinea Pigs Infected with Mycobacterium bovis against the Background of Vaccine Immunity and the Action of Betulin and Its Derivatives
Source: Vaccines (Basel). 2022 Dec 6;10(12):2084. doi: 10.3390/vaccines10122084 (PMC9784198; doi:10.3390/vaccines10122084)
Supplement: Supplementary file 1 [file vaccines-10-02084-s001.zip › vaccines-2035516-supplementary.pdf]

Supplementary Materials

Morphology of Lymphoid Tissue in the Lungs of Guinea Pigs Infected with *Mycobacterium Bovis* against the Background of Vaccine Immunity and the Action of Betulin and Its Derivatives

Ivan N. Koshkin [1], Vasily S. Vlasenko [2,*], Valentina I. Pleshakova [1], Larisa E. Alkhimova [3], Andrey V. Elyshev [3] and Ivan V. Kulakov [3,*]

[1] The Faculty of Veterinary Medicine, Omsk State Agrarian University named after P.A. Stolypin,
2 Institutskaya Ploshchad, Omsk 644008, Russia; in.koshkin@omgau.org (I.N.K.); vi.pleshakova@omgau.org (V.I.P.)
[2] Omsk Agrarian Scientific Center, 26 Koroleva ave., Omsk 644012, Russia
[3] Center of Nature-Inspired Engineering, University of Tyumen, 15a Perekopskaya St., Tyumen 625003, Russia; l.e.alkhim@gmail.com (L.E.A.); a.v.elyshev@utmn.ru (A.V.E.)
* Correspondence: vvs-76@list.ru (V.S.V.); i.v.kulakov@utmn.ru (I.V.K.)

Table of contents

Figure S1. A tuberculous granuloma in contact with the wall of a blood vessel for group one (not vaccinated).
Figure S2. Accumulation of lymphoid tissue near the bronchus for group one (not vaccinated).
Figure S3. Lymph nodule between bronchi and associated vessels for group one (not vaccinated).
Figure S4. A lymph node inculcation in lymphatic vessel for group one (not vaccinated).
Figure S5. Couplings of lymphoid cells around hyperemic vessels for group one (not vaccinated).
Figure S6. Cellular couplings on a transverse section of the connecting vessel for group one (not vaccinated).
Figure S7. Eccentric growth of the lymphoid cells of the coupling, followed by the formation of a follicle for group one (not vaccinated).
Figure S8. Two lymphatic follicles with reactive centers forming near the vessel wall for group one (not vaccinated).
Figure S9. Lymph follicles of varying sizes with eccentric blood vessels (right) for group one (not vaccinated).
Figure S10. Lymphatic follicles with a pronounced reproduction center for group one (not vaccinated).
Figure S11. Compacted area under the pleura as a result of proliferation of epithelioid macrophages for group two (vaccinated with BCG+betulinic acid).
Figure S12. A site compacted as a result of proliferation in the walls of the alveoli of epithelioid cells for group two (vaccinated with BCG+betulinic acid).
Figure S13. Lymphatic follicle near the branches of the large bronchi for group two (vaccinated with BCG+betulinic acid).
Figure S14. A coupling with a small number of lymphocytes around the blood vessel, among the thickened macrophages as a result of proliferation in the walls of the alveoli of epithelioid for group two (vaccinated with BCG+betulinic acid).
Figure S15. A small number of lymphocytes around the blood vessel, which is among the overgrown epithelioid macrophages for group two (vaccinated with BCG+betulinic acid).
Figure S16. Among epithelioid cells, there are a large number of cells with bright red cytoplasm – plasmocytes for group two (vaccinated with BCG+betulinic acid).
Figure S17. A large number of lymphatic follicles with generative centers in the lung tissue for group two (vaccinated with BCG+betulinic acid).
Figure S18. Lymphatic follicle at the branching site of medium-sized bronchi for group three (vaccinated with BCG+betulin).
Figure S19. A blood vessel in the area of the lungs compacted as a result of the proliferation of epithelioid macrophages for group three (vaccinated with BCG+betulin).
Figure S20. The initial stage of formation of a lymphatic follicle near a blood vessel for group three (vaccinated with BCG+betulin).
Figure S21. One of the few follicles with a breeding center for group three (vaccinated with BCG+betulin).

Figure S22. Plasma cells (cells with bright pink cytoplasm) among epithelioid macrophages, as well as near a small lymphatic follicle for group three (vaccinated with BCG+betulin).

Figure S23. Areas of lung tissue compaction of various sizes for group four (vaccinated with BCG+betulonic acid).

Figure S24. A small accumulation of lymphoid cells near a medium-sized bronchus for group four (vaccinated with BCG+betulonic acid).

Figure S25. Cellular couplings of a small number of lymphocytes around a blood vessel in the area of proliferation of epithelioid macrophages for group four (vaccinated with BCG+betulonic acid).

Figure S26. Plasma cells (cells with bright pink cytoplasm) in the walls of the alveoli for group four (vaccinated with BCG+betulonic acid).

Figure S27. The small size follicle without a pronounced reproduction center for group four (vaccinated with BCG+betulonic acid).

Figure S28. Accumulation of lymphoid cells near the bronchus and artery for group five (vaccinated with BCG).

Figure S29. A small collection of lymphoid cells in the form of a nodule in the peribronchial tissue for group five (vaccinated with BCG).

Figure S30. Lymph follicles maintain contact with blood vessels for group five (vaccinated with BCG).

Figure S31. A large lymphatic follicle located between the bronchus and the artery for group five (vaccinated with BCG).

Figure S32. A few perivascular couplings show no evidence of lymphatic follicle formation for group five (vaccinated with BCG).

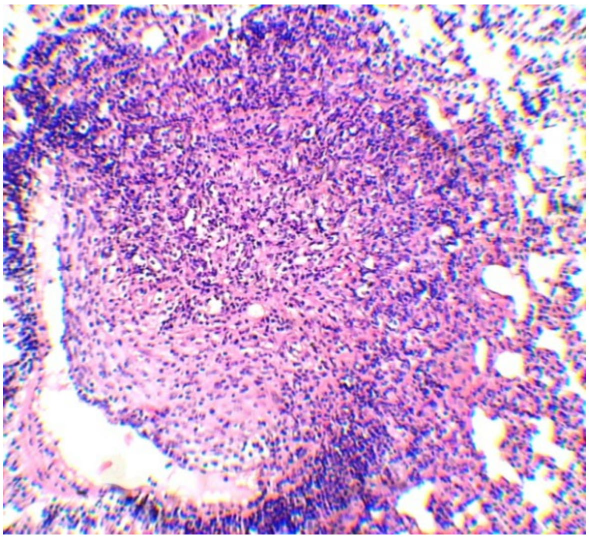

Figure S1. A tuberculous granuloma in contact with the wall of a blood vessel for group one (not vaccinated). Only proliferative processes and infiltration by lymphocytes are observed.

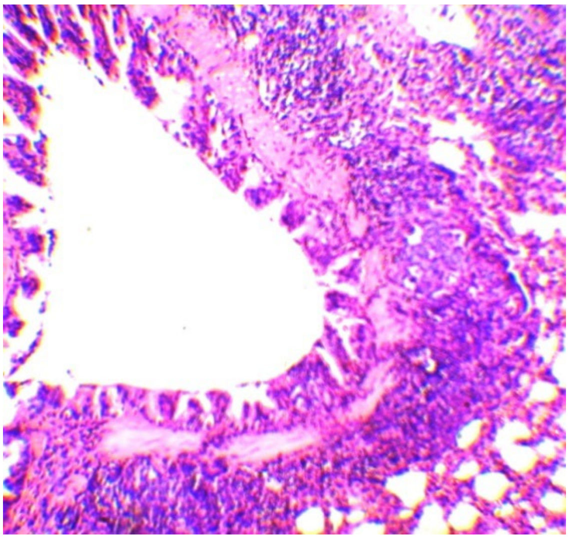

Figure S2. Accumulation of lymphoid tissue near the bronchus for group one (not vaccinated).

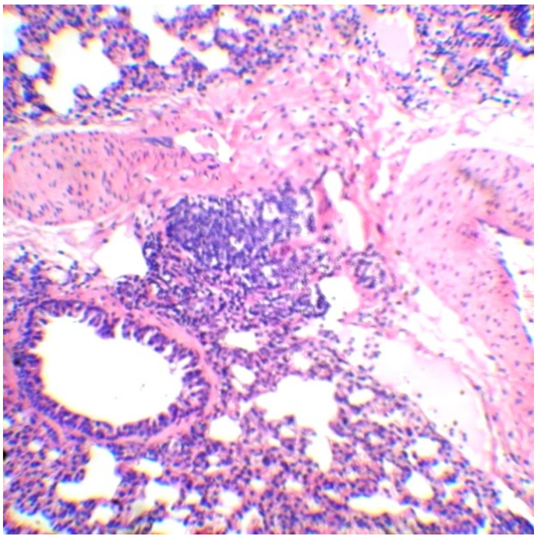

Figure S3. Lymph nodule between bronchi and associated vessels for group one (not vaccinated). On the right and on the left next to the site of ulceration of the vessels. The dilated vessel of the bleeding vessel is clearly visible in the right corner, where it passes through the inflammation of the vascular vessel.

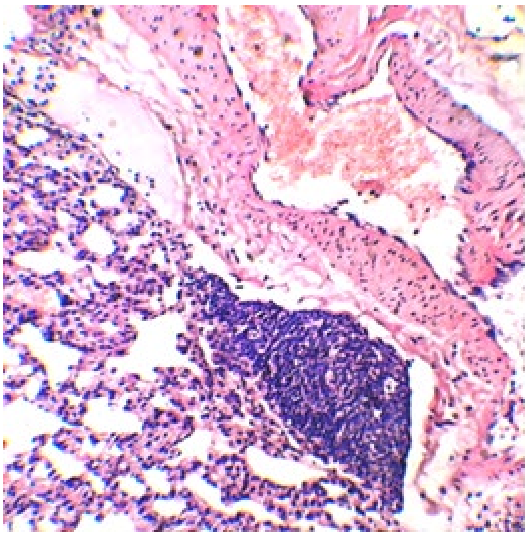

Figure S4. A lymph node inculcation in lymphatic vessel for group one (not vaccinated).

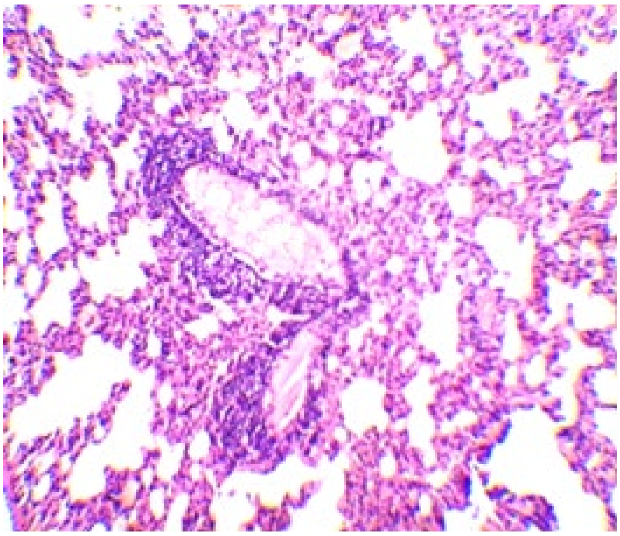

Figure S5. Couplings of lymphoid cells around hyperemic vessels for group one (not vaccinated).

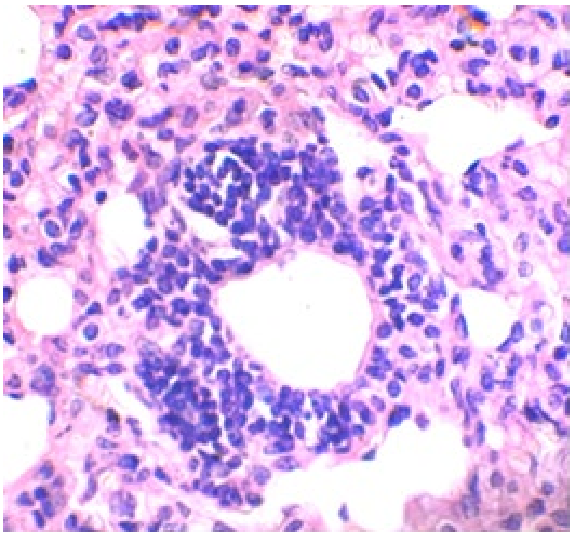

Figure S6. Cellular couplings on a transverse section of the connecting vessel for group one (not vaccinated). Thickening of the walls of the alveoli due to the proliferation of epithelioid cells.

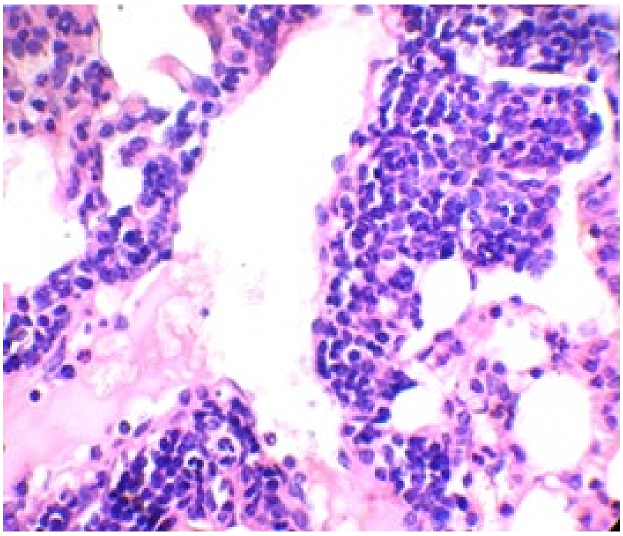

Figure S7. Eccentric growth of the lymphoid cells of the coupling, followed by the formation of a follicle for group one (not vaccinated).

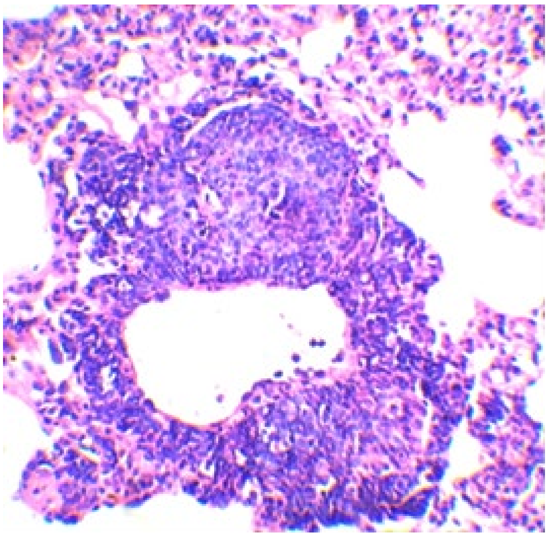

Figure S8. Two lymphatic follicles with reactive centers forming near the vessel wall for group one (not vaccinated).

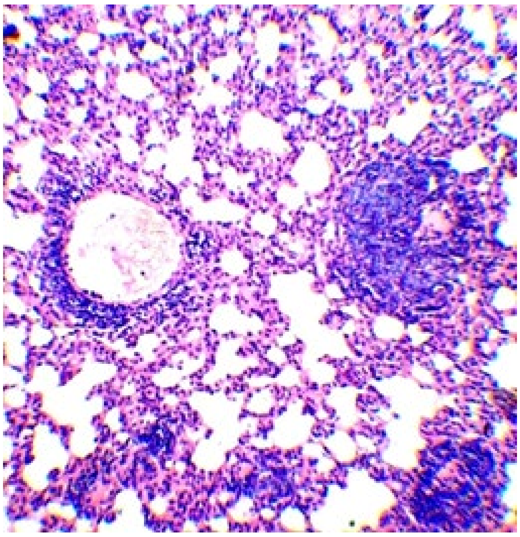

Figure S9. Lymph follicles of varying sizes with eccentric blood vessels (right) for group one (not vaccinated).

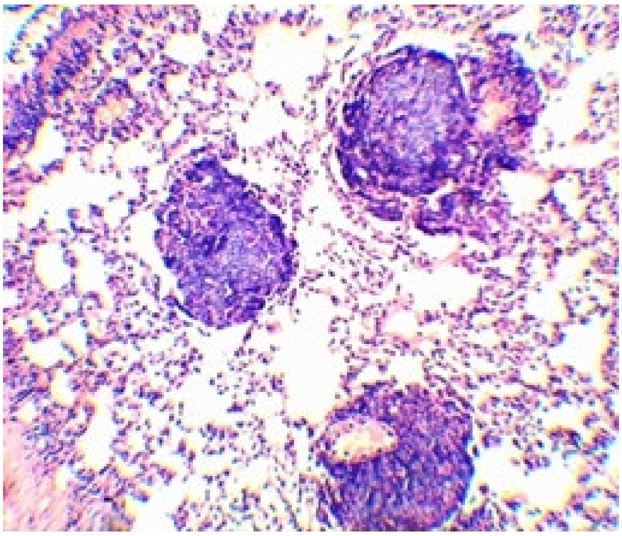

Figure S10. Lymphatic follicles with a pronounced reproduction center for group one (not vaccinated). In the lower follicle lies an enlarged blood vessel.

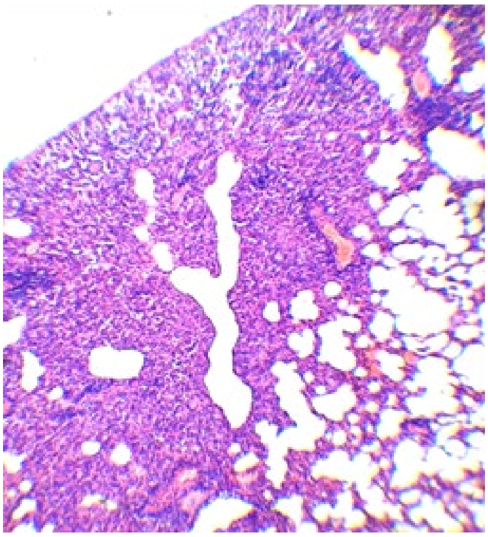

Figure S11. Compacted area under the pleura as a result of proliferation of epithelioid macrophages for group two (vaccinated with BCG+betulinic acid). Around the vessels there are couplings of lymphocytes.

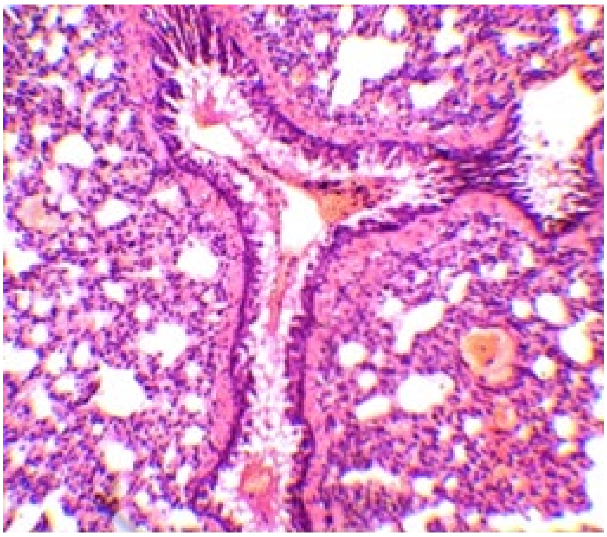

Figure S12. A site compacted as a result of proliferation in the walls of the alveoli of epithelioid cells for group two (vaccinated with BCG+betulinic acid). There is no accumulation of lymphoid cells around the bronchi.

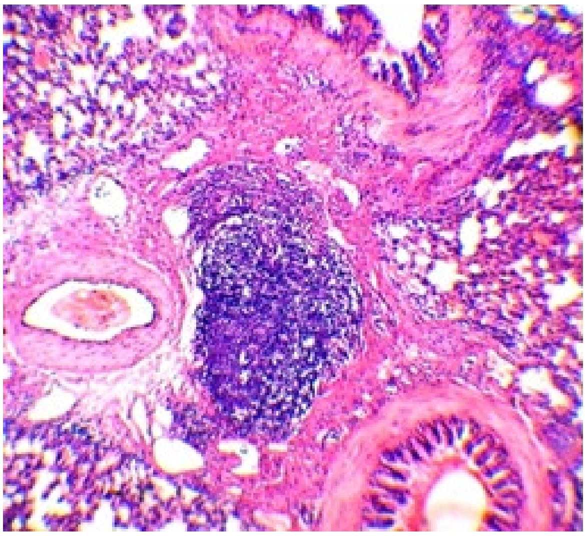

Figure S13. Lymphatic follicle near the branches of the large bronchi for group two (vaccinated with BCG+betulinic acid).

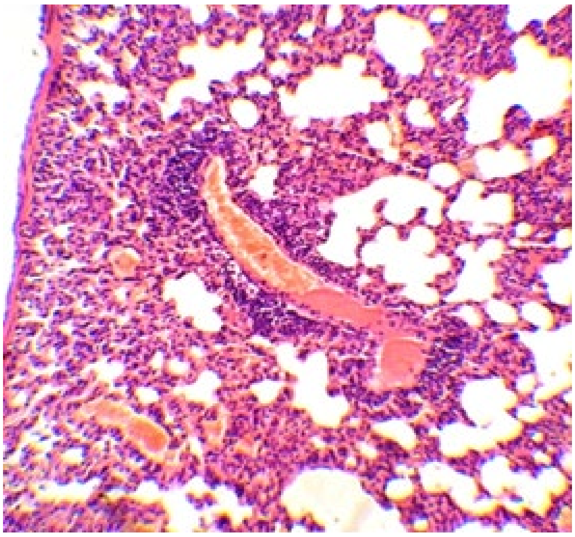

Figure S14. A coupling with a small number of lymphocytes around the blood vessel, among the thickened macrophages as a result of proliferation in the walls of the alveoli of epithelioid for group two (vaccinated with BCG+betulinic acid).

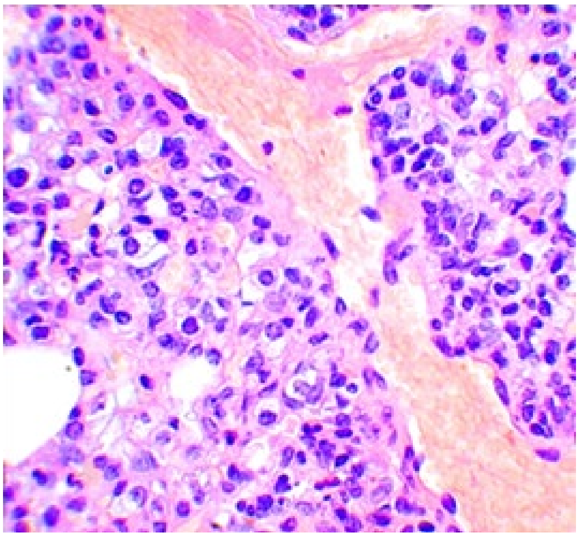

Figure S15. A small number of lymphocytes around the blood vessel, which is among the overgrown epithelioid macrophages for group two (vaccinated with BCG+betulinic acid).

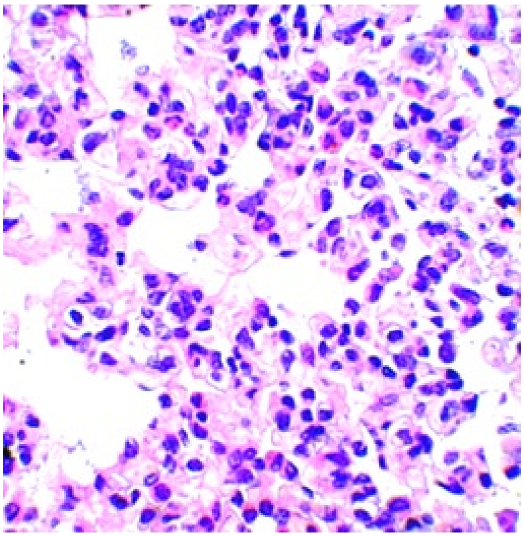

Figure S16. Among epithelioid cells, there are a large number of cells with bright red cytoplasm – plasmocytes for group two (vaccinated with BCG+betulinic acid).

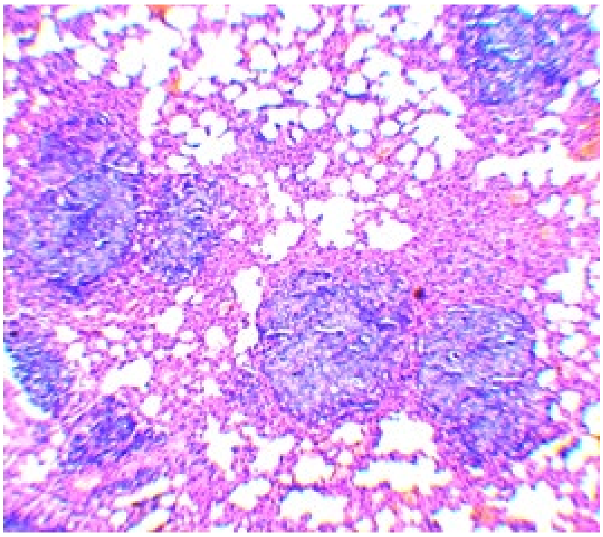

Figure S17. A large number of lymphatic follicles with generative centers in the lung tissue for group two (vaccinated with BCG+betulinic acid). Around them areas compacted as a result of proliferation of epithelioid cells. In the follicles, reproductive centers are expressed.

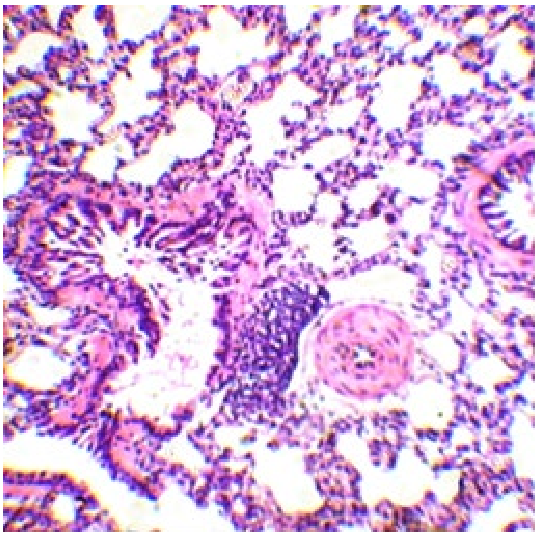

Figure S18. Lymphatic follicle at the branching site of medium-sized bronchi for group three (vaccinated with BCG+betulin). It lies between the bronchi and adjacent vessels.

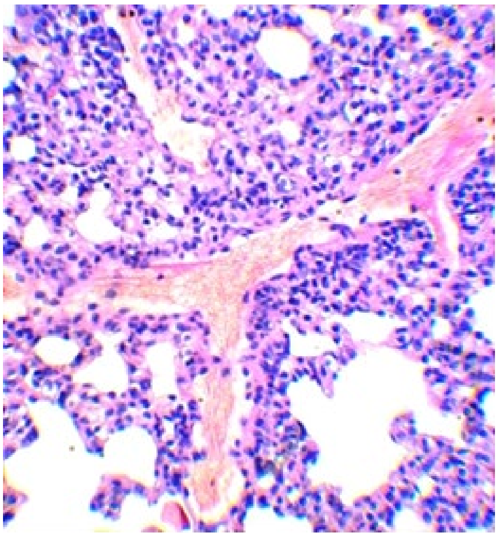

Figure S19. A blood vessel in the area of the lungs compacted as a result of the proliferation of epithelioid macrophages for group three (vaccinated with BCG+betulin). Near it there is a slight accumulation of lymphoid cells.

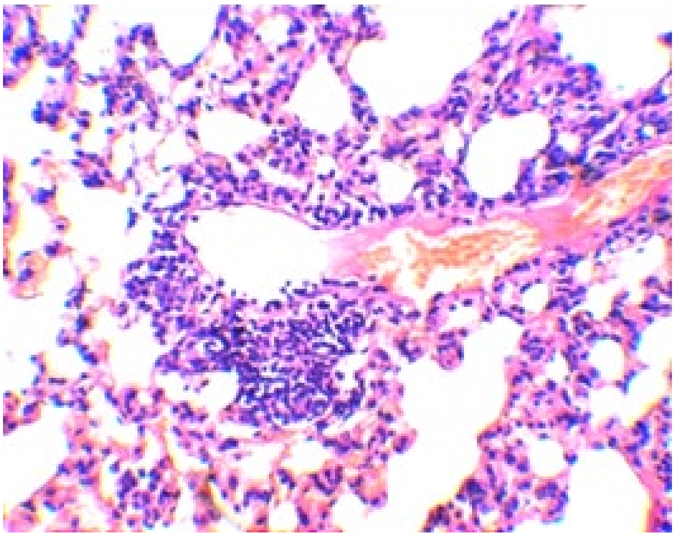

Figure S20. The initial stage of formation of a lymphatic follicle near a blood vessel for group three (vaccinated with BCG+betulin acid).

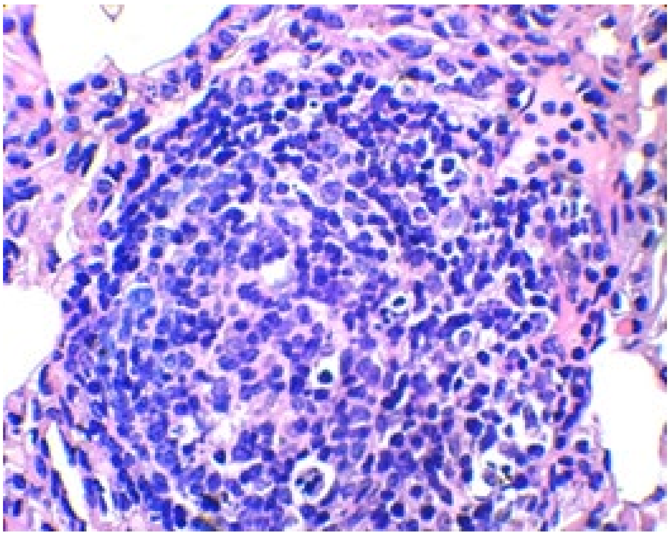

Figure S21. One of the few follicles with a breeding center for group three (vaccinated with BCG+betulin). In the upper right corner of the lymphatic vessel with lymphocytes. On the right is a plasma cell.

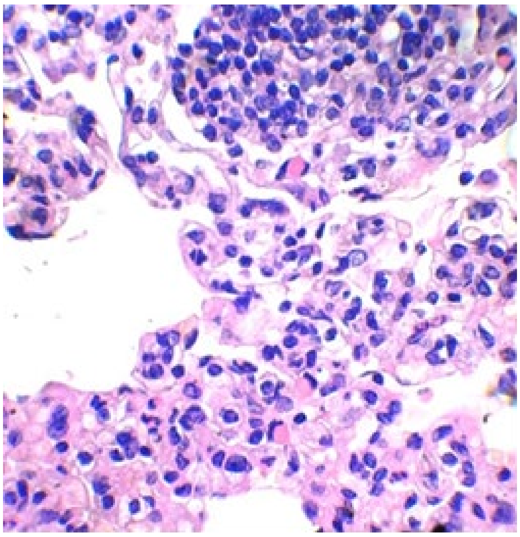

Figure S22. Plasma cells (cells with bright pink cytoplasm) among epithelioid macrophages, as well as near a small lymphatic follicle for group three (vaccinated with BCG+betulin).

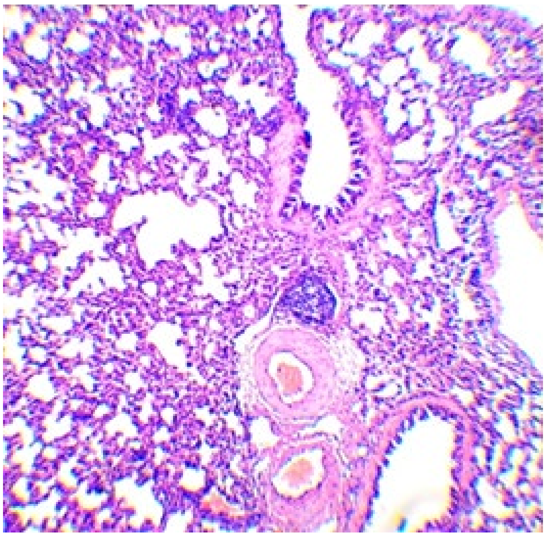

Figure S23. Areas of lung tissue compaction of various sizes for group four (vaccinated with BCG+betulonic acid). Near the medium-sized bronchi and blood vessels there is a lymphatic follicle. There are no diffuse accumulations of lymphoid cells near the walls of the bronchi.

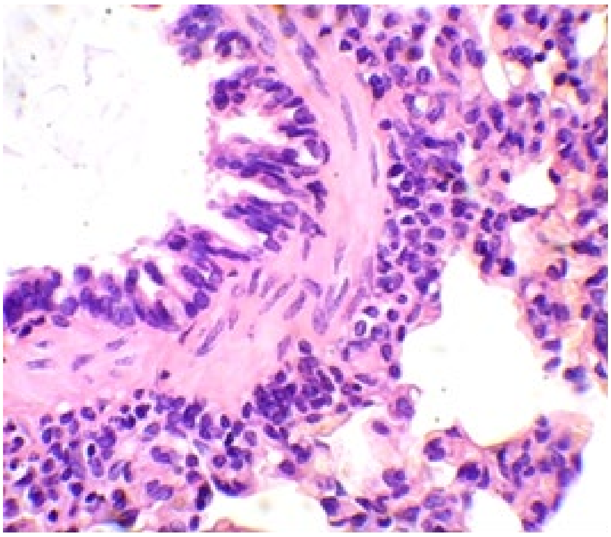

Figure S24. A small accumulation of lymphoid cells near a medium-sized bronchus for group four (vaccinated with BCG+betulonic acid).

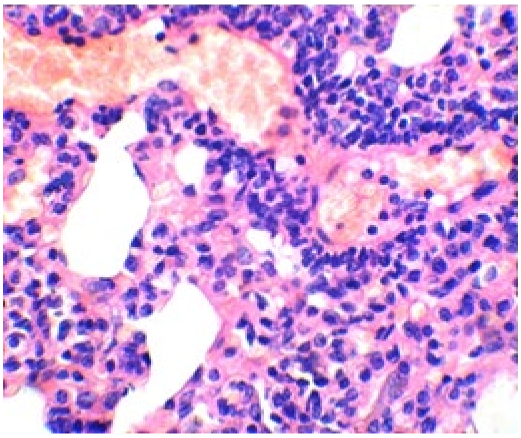

Figure S25. Cellular couplings of a small number of lymphocytes around a blood vessel in the area of proliferation of epithelioid macrophages for group four (vaccinated with BCG+betulonic acid).

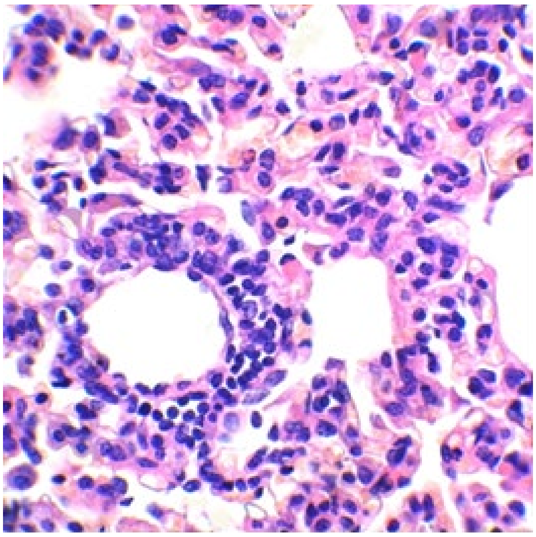

Figure S26. Plasma cells (cells with bright pink cytoplasm) in the walls of the alveoli for group four (vaccinated with BCG+betulonic acid). Lymphocyte clutch around a small vessel.

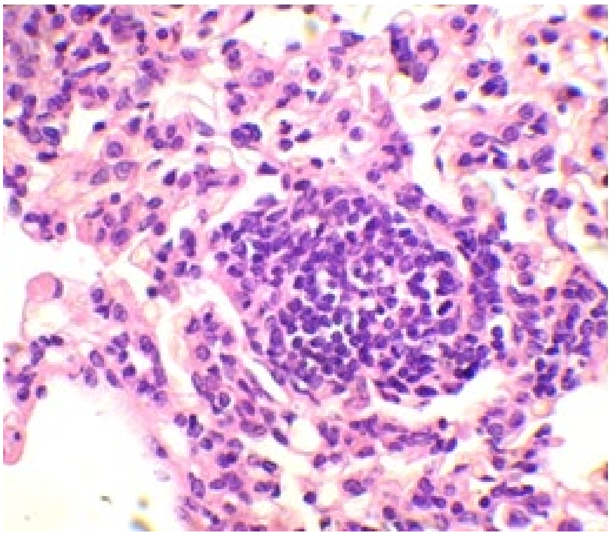

Figure S27. The small size follicle without a pronounced reproduction center for group four (vaccinated with BCG+betulonic acid). At the left edge of the figure in the center are two plasma cells.

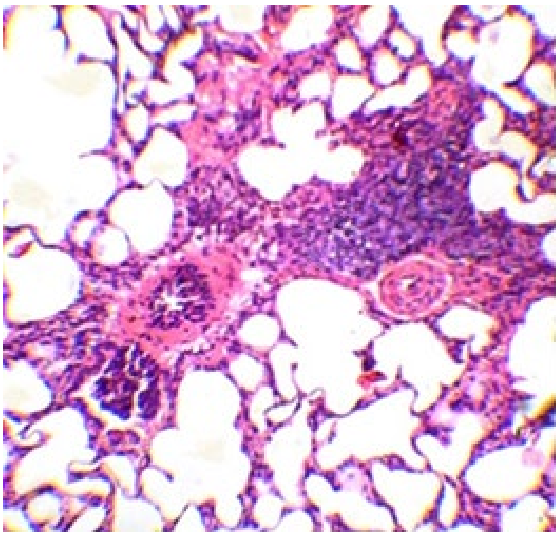

Figure S28. Accumulation of lymphoid cells near the bronchus and artery for group five (vaccinated with BCG).

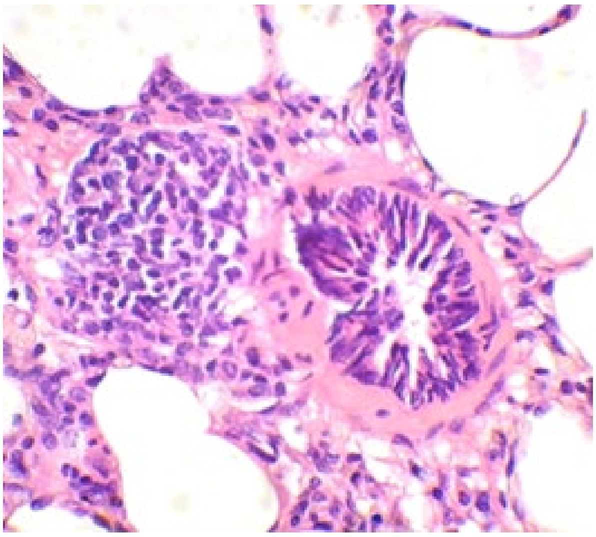

Figure S29. A small collection of lymphoid cells in the form of a nodule in the peribronchial tissue for group five (vaccinated with BCG).

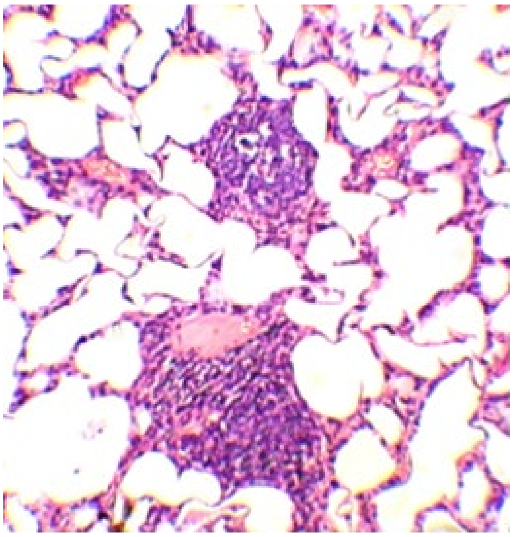

Figure S30. Lymph follicles maintain contact with blood vessels for group five (vaccinated with BCG). There are no breeding centers and reactive centers in them.

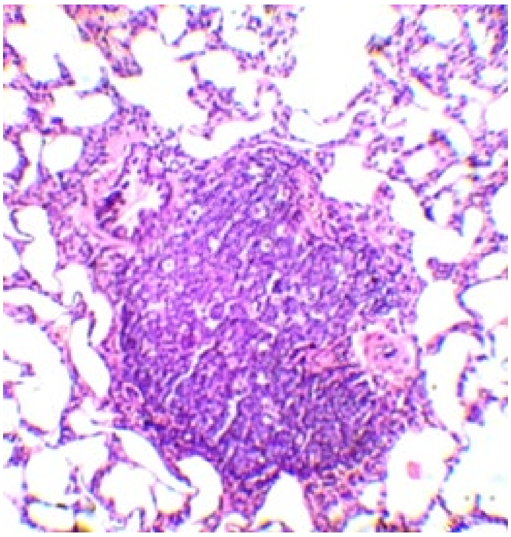

Figure S31. A large lymphatic follicle located between the bronchus and the artery for group five (vaccinated with BCG).

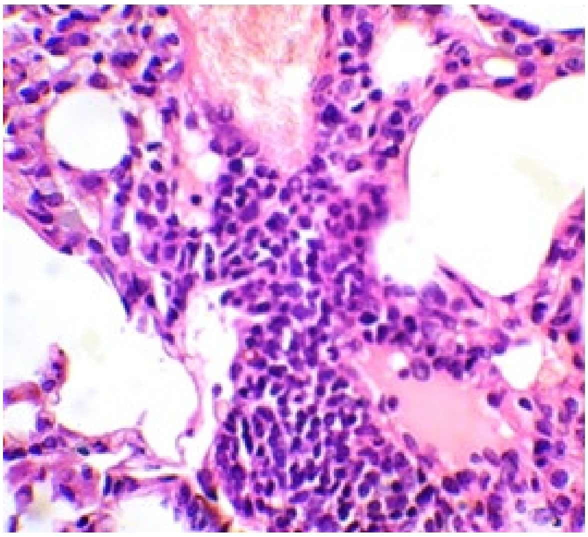

Figure S32. A few perivascular couplings show no evidence of lymphatic follicle formation for group five (vaccinated with BCG).
